# Particularities of Neurological Manifestations in Adult T-Cell Leukemia/Lymphoma: Need for a Multidisciplinary Approach—A Narrative Review

**DOI:** 10.3390/medicina58111553

**Published:** 2022-10-28

**Authors:** Iuliana Iordan, Minodora Onisâi, Ana-Maria Vlădăreanu, Cristina Mambet, Elena Cristina Marinescu, Raluca Nistor, Horia Bumbea

**Affiliations:** 1 Department of Hematology, “Carol Davila” University of Medicine and Pharmacy, Emergency University Hospital of Bucharest, 050098 Bucharest, Romania; 2 Department of Medical Semiology and Nephrology, “Carol Davila” University of Medicine and Pharmacy, 050474 Bucharest, Romania; 3 Department of Virology, “Stefan S. Nicolau” Institute, 030304 Bucharest, Romania; 4 Department of Neurology, “Carol Davila” University of Medicine and Pharmacy, Emergency University Hospital of Bucharest, 050098 Bucharest, Romania

**Keywords:** ATL, HAM/TSP, neurological manifestations, CNS involvement, neurolymphomatosis, infection

## Abstract

ATL is a rare but a highly aggressive T-cell neoplasm associated with human T-cell leukemia virus-1 (HTLV-1) infection. Human T-cell lymphotropic virus type-1 (HTLV-1) is a oncogenic retrovirus responsible for the development of adult T-cell leukemia (ATL), but also for other non-malignant diseases, such as HTLV-1-associated myelopathy/tropical spastic paraparesis (HAM/TSP). HTLV-1 has a higher prevalence in Japan, the Caribbean, South America, intertropical Africa, Romania, and northern Iran. ATL patients can have an extensive spectrum of neurological manifestations. Numerous factors can be implicated, such as central nervous system infiltrates, neurolymphomatosis, complications to medication or allogeneic stem cell transplantation, HAM/TSP, infections, metabolic disturbances. The neurological complications are not always easy to recognize and treat. Thus, this review underlines the necessity of a multidisciplinary approach in ATL patients with neurological symptomatology.

## 1. Introduction

ATL is a rare and typically severe lymphoproliferative disorder caused by infection with human T-cell lymphotropic virus (HTVL-1), characterized by the proliferation of peripheral T-cells [1].

Human T-cell lymphotropic virus (HTLV-1) is an oncogenic retrovirus that causes adult T-cell leukemia (ATL) as well as non-malignant disorders such as HTLV-1-associated myelopathy/tropical spastic paraparesis (HAM/TSP), uveitis, and infective dermatitis [1]. HTLV-1 has a high prevalence in Japan, the Middle East, Central and South America, the Caribbean Islands, several African countries, the Solomon Islands, Vanuatu, central Australia, and Romania [1,2,3].

ATL was fist described as a distinctive pathology in 1977 in Japan [4]. HTLV-1 carriers have a 3–5% lifetime risk of developing ATL [5]. ATL usually develops in adults after 20–30 years, especially when exposed in childhood [5]. In 1991, Shimoyama et al., classified ATL into four categories, namely smoldering, chronic, lymphomatous, and acute [6]. The last two, as well as the unfavorable chronic one, are classified as aggressive types [6]. Despite advancements in treatment, the prognosis for aggressive types of ATL remains poor, with a 4-year survival rate of 16.8–26.6%, whereas it is approximately 60% for the indolent types [7]. Allogeneic stem cell transplantation is the only potentially curative option and improves prognosis in aggressive types of ATL [8,9].

ATL exhibits particularities regarding neurological manifestations, especially regarding central nervous system (CNS) invasion and the possible association with HAM/TSP in ATL patients. ATL is more likely to infiltrate CNS compared to other T-cell lymphomas [10]. Many cases of concurrent HAM/TSP were documented [11,12,13,14,15,16,17,18,19,20,21,22,23,24,25,26,27,28,29,30,31,32,33], despite the fact that both diseases are uncommon, raising the question of whether the connection is meaningful or merely coincidental [34]. Furthermore, various additional causes of neurological symptoms might be implicated, including neurolymphomatosis [27,35,36,37,38], treatment-related complications [39], particularly allogeneic stem cell transplantations (allo-HSCT) [40,41], hypercalcemic crisis [42,43,44], and CNS infections [45,46,47]. Because the symptomatology and even laboratory and imagistic results may be similar, it is critical to appropriately diagnose and treat the neurological complication. This literature review presents the summary of the most relevant neurological complications in ATL patients.

## 2. CNS Involvement in ATL

ATL patients have a higher risk of CNS involvement than other T-cell lymphoma patients [10]. The incidence of CNS disease in ATL ranges from 10% to 25%, whereas it is only 6.4% in the other types [10,48,49]. Furthermore, this incidence may be underestimated, as CNS involvement was discovered during postmortem brain examination in asymptomatic patients [49]. Additionally, even if there is CNS involvement, cerebrospinal fluid (CSF) and imaging studies may be negative [50,51]. CNS involvement is more typical in lymphoma and acute types [48,49,51,52]. When CNS is involved, the systemic disease is almost always present [51]. There are only a few reports of isolated CNS lymphoma in ATL [31,50,53,54,55,56].

Clinical manifestations are similar among different types of lymphomas, with the differences arising from the affected CNS regions. Muscle weakness, altered mental status, paresthesia, headaches, and bladder dysfunction are some of the initial symptoms [49]. Other manifestations include neurological deficits, cranial nerve palsies, visual impairments, altered consciousness, psychiatric conditions, behavioral abnormalities, and seizures [49,51].

National Comprehensive Cancer Network guidelines recommend CSF analysis and/or imaging by CT (computed tomography) scan or MRI (magnetic resonance imaging) for all ATL patients with acute or lymphoma type even if neurological manifestations are absent [57]. In rare cases, a biopsy is required to confirm the diagnosis. Lumbar puncture with CSF analysis should always be performed in all cases of acute and lymphoma-type ATL after the first round of chemotherapy [9]; increased cell counts, high protein levels, and hypoglycorrhachia are common findings when CNS is affected [49]. Three imagistic categories—definite, probable, and other abnormal—are used to categorize CNS lesions in ATL [52]. Parenchymal masses, with or without enhancement, and leptomeningeal enhancement are the most common ATL-related findings (definite and probable). The majority of these lesions are located in the cerebral cortex, corpus callosum, basal ganglia, thalamus, and white matter [52]. The biopsies taken from the brain lesions revealed clusters of tumoral cells infiltrating the perivascular spaces, parenchyma, and leptomeninges. In the spinal cord, tumor cells invade the leptomeninges and grey matter [52].

CNS prophylaxis is strongly suggested for all patients with acute or lymphoma-type ATL [57]. The 2019 Revised Adult T-Cell Leukemia-Lymphoma International Consensus Meeting Report recommends intrathecal chemotherapy for asymptomatic patients following the first cycle of treatment as well as high-dose methotrexate-based regimens or intrathecal chemotherapy for patients who have CNS disease upon diagnosis [9].

The utility of allo-HSCT for ATL patients with CNS disease is still up for debate. According to current recommendations, a conditioning regimen which includes drugs that can cross the blood-brain barrier, such as Thiotepa should be considered [9]. Additionally, CNS disease patients may benefit from graft-versus-leukemia effect. When grade III-IV acute or extensive chronic graft-versus-host disease (GVHD) was present, positive outcomes were reported [58]. Three out of four patients with severe acute or extensive chronic GVHD among ATL patients with CNS illness who undergo allo-HSCT survived for at least three years following transplantation [58]. The outcome of allo-HSCT seems to be affected by CNS involvement. Before transplantation, patients with CNS disease who had reached complete remission had similar outcomes to those without CNS infiltration, while those who had persistent CNS infiltration had shorter overall survival and a greater risk of relapse or progression one year later [59].

Low efficiency and high transplant-related mortality point to the necessity for the implementation of safer and more effective methods. Intrathecal donor lymphocyte infusion was used in CNS relapses of other diseases, such as acute myeloid leukemia, chronic myeloid leukemia, T-cell leukemia, and T-cell lymphoma [60]. However, outside of clinical trials, this strategy is still unproven and is not advised [60].

## 3. Neurolymphomatosis

Neurolymphomatosis is an uncommon and challenging neurological complication defined by the invasion of malignant lymphocytes into the nerves [61,62]. It can occur in non-Hodgkin lymphoma and acute lymphoid leukemia at any stage during evolution. It can be the first indication of lymphoma or be associated with recurrence or progression of the disease [62].

Neurolymphomatosis is an uncommon entity in T-cell lymphoma. As reported by Grisariu et al., only 4% of neurolymphomatosis cases are associated with T-cell lymphoma [62]. To our best knowledge, only five cases of neurolymphomatosis in ATL patients have been reported in the literature (Table 1) [27,35,36,37,38].

The clinical presentation of neurolymphomatosis is heterogeneous since it typically affects numerous distinct nerves [27,36,38,62]. Peripheral, spinal, and cranial nerves can all be involved [62]. The most frequently reported symptoms in afflicted areas are pain, paresthesia, and weakness [27,35,37,38,63]. Symptoms can occasionally resemble Guillain-Barre syndrome or chronic inflammatory demyelinating polyneuropathy [64,65].

Imaging methods including ultrasonography, MRI, and fluorodeoxyglucose-positron emission tomography (FDG-PET) may show high sensitivity for neurolymphomatosis. However, in most cases, a nerve biopsy with a histological and immunohistochemical examination is required to establish the diagnosis of neurolymphomatosis [62,66]. The most commonly reported MRI abnormality is diffuse or nodular enlargement and enhancement of the neural structure [27,36,62]. These changes, however, are not specific to neurolymphomatosis [65]. FDG-PET is a more sensitive technique that can be helpful for diagnosis and post-treatment monitoring [62,67]. When a nerve is affected by neurolymphomatosis, FDG-PET demonstrates increased uptake [27,38,67]. These approaches, however, are not always accessible. Nerve ultrasound is a more convenient technique. Therefore, it should be considered in those patients with lymphoma and focal neuropathy. The ultrasonographic signs of neurolymphomatosis include enlarged nerves, structural anomalies, and increased vascularity [27,66].

**Table 1:** Published cases of neurolymphomatosis associated with ATL [27,35,36,37,38,68].

No.	Authors	Year	Age	Sex	Type of ATL	Involved Nerves	Manifestations	CSF	Imagistics	Biopsy
1	Kuroda Y et al. [35]	1989	60	M	N/A	N/A	Sensorimotor polyneuropathy, persistent severe pain	N/A	N/A	Autopsy: infiltration of peripheral nerves by leukemia
2	Umehara et al. [27]	2007	56	F	chronic	Cervical nerve roots Radial, median, ulnar nerves	Progressive muscle weakens upper limbs Paresthesia left limb	↑ anti-HTLV-1 antibody titer	MRI: enhancement and enlargement of the involved nerves. Ultrasonography: enlargement of the involved nerves. FDG-PET: increased uptake in the involved nerves	N/A
3	Liang J et al. [36]	2014	49	M	N/A	Bilateral involvement of cranial nerves: III, V, VI, VII, VIII	Multiple cranial neuropathy	normal	MRI: enhancement and enlargement of the involved nerves	Nerve biopsy: diffuse infiltration of nerve fascicles by pleomorphic lymphocytes
4	Yoshimitsu M et al. [37]	2015	74	M	smoldering	Sural nerve	Muscle weakness of the extremities, painful paresthesia	N/A	FDG-PET: no signs of neurolymphomatosis	Atypical lymphocytes infiltrating perineural connective tissue and subperinerurium
5	Ono Y et al. [38]	2017	48	F	acute	Sciatic, iliac, recurrent laryngeal nerves Cervical nerve plexuses	Feet dysesthesia, right eye diplopia	+ Atypical lymphocytes	MRI: no signs of neurolymphomatosis. FDG-PET: significant uptake in involved nerves	N/A

Abbreviations: ATL = adult T cell leukemia; HTLV-1 = human T-cell lymphotropic virus; N/A = not available; MRI = magnetic resonance imaging; FDG-PET = fluorodeoxyglucose-positron emission tomography; M = male; F = female; CSF = cerebrospinal fluid.

CSF analysis can be useful in the diagnosis of neurolymphomatosis, although the results may be inconclusive [27,36,61]. It frequently shows increased protein, hypoglycorrhachia, and an elevated cell count, particularly abnormal lymphocytes [38,61]. Flow cytometry can further categorize the abnormal cells [61]. As shown in Table 1, abnormal cytology was seen in only one out of the three ATL cases in which this analysis was performed or available [27,36,38]. When imagistic studies and CSF analysis are negative, nerve biopsy can be a viable option in selected cases. The latter was the most accurate test for diagnosis, being suggestive of neurolymphomatosis in 88% of cases [62]. However, a patchy infiltration might provide false negative results. Finding the optimal site using imagistic (FDG-PET, nerve ultrasonography) could reduce the false negatives [65,66,67]. In neurolymphomatosis, the histological exam describes lymphocytic infiltration of the peripheral nerve. In addition, immunohistochemical studies should accompany the histological ones in order to diagnose the type of lymphoma or leukemia [65].

There is no consensus regarding treatment for neurolymphomatosis. Chemotherapy alone or in combination with radiation or intrathecal therapy are the main forms of treatment [61,62,63]. Intensive chemotherapy with drugs that can cross the blood–brain barrier, such as methotrexate, thiotepa, and cytarabine, is widely applied [62,63]. However, the clinical outcome is typically poor [63].

## 4. HAM/TSP

### 4.1. Overview of HAM/TSP

HAM/TSP is a rare chronic neurodegenerative disorder. Only a minority of HTLV-1 carriers will develop HAM/TSP. The estimated lifetime risk of HAM/TSP among HTLV-1 carriers is approximately 0.18–1.8% and is higher in women [69,70].

The diagnosis of HAM/TSP is based on the presence of characteristic neurological features in an HTLV-1-positive patient. The diagnosis criteria were defined by the World Health Organization in 1989 (Table 2) [71]. Frequently, a single sign or symptom may be the only evidence of early HAM/TSP. It is necessary to distinguish this disease from others with similar manifestations, such as multiple sclerosis [72]. MRI studies show high sensitivity in detecting anomalies that could be attributed to other disorders. Thoracic cord atrophy and white matter anomalies located preferentially periventricular are typical but not specific findings in HAM/TSP patients [72,73,74]. Patients with a more severe disease tend to have a higher number of more extensive white matter lesions [72]. Peripheral neuropathy is frequent among HAM/TSP patients but is often asymptomatic. Electrophysiological studies reported several abnormalities in almost half of the patients, namely axonal and demyelinating sensory and motor neuropathy [75].

The clinical course of the disease is different among individuals. The neurological manifestations aggravate in a variable period. There are three types of evolutions, depending on the rate of progression, namely very slow, slow, and rapid progressors. Studies describe several biomarkers that correlate with disease activity, such as proviral load in peripheral blood, cell counts, anti-HTLV-1 antibody titer, protein, neopterin, and C-X-C motif chemokine 10 (CXCL10) levels in CSF [78,79]. In 2018, Sato et al., proposed the classification criteria for disease activity based on the rapidity of neurological degradation and two biomarkers: neopterin and CXCL10 levels in CFS. The authors considered the rapid progressors of those who developed Osame motor disability score (OMDS) (Table 3) grade 5 or higher in two years since diagnosis. The very slow progressors developed OMDS grade 3 or lower in 10 years since diagnosis. Those who did not meet the criteria for either of these two classes were considered slow progressors. The division into three categories predicts the long-term functional prognosis. The median time of progression from OMDS grade 2 to 6 was four years for rapid progressors, nine years for slow progressors, and 35 years for very slow progressors (*p* < 0.0001). Biomarkers analysis demonstrated significant differences in neopterin and CXCL10 levels in CSF between all three groups. Based on the clinical course and biomarkers, the authors proposed the classification criteria for disease activity, as depicted in Table 4 [80].

Determining the disease activity and, thus, estimating the risk of progression assist in avoiding excessive treatment. Two types of therapies can be used for HAM/TSP patients: symptomatic and disease-modifying agents. The symptomatic therapies are muscle relaxers, analgesics, and physiotherapy [82]. Although they improve the quality of life, they do not delay the progression [83]. The disease-modifying agents (corticosteroids, antiretroviral therapy, cytotoxic agents, interferon-alpha, plasma exchange, and other immunomodulatory agents) should be considered for all patients with HAM/TSP, regardless of the severity and disease activity [83,84]. The most commonly used drugs are corticosteroids: induction with high-dose pulse methylprednisolone or high-dose prednisolone, followed by maintenance with low-dose corticosteroids [83]. Other drugs, such as interferon-alpha, sodium valproate, antiretroviral therapy, and anti-CCR4 monoclonal antibody, are not indicated outside clinical trials [83,85]. Combined therapy shows advantages in reducing the symptoms and disease-activity biomarkers. Triple therapy with interferon-alpha, sodium valproate, and prednisolone was proven efficient in improving symptomatology, except for the urinary one, and decreasing HTLV-1 proviral load, HTLV-1 antibody titer, HBZ (HTLV-1 basic leucine zipper factor), and Tax (trans-activator x) expression [84].

### 4.2. Common Points in ATL and HAM/TSP Pathogenesis

There is no consensus on how ATL or HAM/TSP develop in HTLV-1 carriers. HTLV-1 proviral load is considered one of the determining factors in HTLV-1-associated diseases. Patients with ATL have the highest levels of HTLV-1 proviral load, followed by those with HAM/TSP. In both cases, the mean value was higher than in healthy carriers [86]. However, the proviral load alone cannot always predict the outcome. Tax and HBZ are oncogenes expressed by the HTLV-1 genome. Tax and HBZ expressions are higher in ATL and HAM/TSP than in asymptomatic carriers [87,88]. However, Tax expression is lost in a significant proportion of ATL cases, while in HAM/TSP, it plays an essential role by promoting the proliferation of infected cells and dysregulation of immune cells. Then, HBZ is always expressed by ATL cells, enabling proliferation of malignant cells and protecting them against apoptosis by evading the host immune response [88,89]. A more recent approach involves using gene expression analysis in order to identify the pathways interested in the development of each disease. It found that viral and immune-related pathways were activated in both HAM/TSP and ATL, while cancer pathways were activated in ATL and neurological ones in HAM/TSP [90].

### 4.3. Coexistence of HAM/TSP and ATL

Concomitant occurrence of HAM/TSP and ATL is rarely reported, with earlier data suggesting that it is purely coincidental. Nagasaka M et al., analyzed ATL cases in HAM/TSP patients from the Japanese HAM-net registry. The authors observed a higher incidence of ATL in HAM/TSP patients than in healthy HTLV-1 carriers, of 3.81/1000 persons per year, compared to 0.6–1.3/1000 persons per year. These findings suggest a higher risk for ATL in HAM/TSP patients. Therefore, it will be helpful to identify the at-risk patients in order to provide early intervention and improve prognosis. The authors performed flow cytometry, Southern blotting, and targeted sequencing to identify higher-risk patients. First, the authors identified, by flow cytometry, 37 out of 218 patients with HAM/TSP and a high prevalence of HTLV-1 infected cells (>25% CD4+ cells positive for CADM1 (Cell Adhesion Molecule 1)). Twenty-seven out of the 37 cases were evaluated with Southern blotting and 21 with targeted sequencing. Two patients developed ATL during the median period of observation of 4.93 years. The first case had a CADM1+CD7- dominant pattern. The second case initially had a CADM1+CD7dim dominant pattern, but it changed to CADM1+CD7- during evolution. Both had a clonal band of HTLV-1 and “high-risk” somatic mutations at a high variant allele frequency (PLCG1, POT1, TET2, GATA3) [34].

Most data about the association of HAM/TSP come from case reports. A total of 26 cases of HAM/TSP associated with ATL were identified in the literature (Table 5). To our knowledge, the first case of a patient with both ATL and HAM/TSP was reported in 1986 by Bartholomew et al., (Table 5, case no.1) [11]. The clinical course was variable. In most cases, ATL was diagnosed after a long evolution of HAM/TSP. Four cases were diagnosed with ATL before the onset of HAM/TSP symptoms (no. 7, 9, 15, 22), and three were diagnosed with both diseases concurrently (no. 10, 19, 20). The age at ATL diagnosis was between 16 and 72-year-old (Table 5). Case no. 19, as far as we know, is the only report regarding the association of HAM/TSP with ATL in adolescents. A 16-year-old girl was diagnosed with smoldering ATL and HAM/TSP after a history of infective dermatitis associated with HTLV-1 for at least four years [26].

We noted the association of two separate neurological disorders in two cases reported by Umehara et al. [27] and Takeda et al. [31]. Patient no. 21 was diagnosed with chronic ATL and developed muscle weakness and paresthesia in the upper limbs. As depicted in Table 1, the CSF analysis and imagistic studies established the diagnoses of both HAM/TSP and neurolymphomatosis. Case no. 24 presented with ATL in the CNS after a 20-year history of HAM/TSP. The differential diagnosis was challenging in this particular case, as no other typical symptoms and signs for ATL were present. The cranial nerve symptoms, which are not usually observed in HAM/TSP patients, indicate another cause. The authors performed MRI and CSF analyses. The MRI revealed the presence of multiple lesions in the frontal cerebral white matter, the corpus callosum, and the pons. The CSF revealed higher sIL-2R levels, proviral load, and CADM1+CD7- cells than in the peripheral blood. The evolution was dismal, with the patient dying six days after admission [31]. The neurological manifestations of HAM/TSP can conceal ATL progression with CNS involvement or neurolymphomatosis. Thus, one should consider other etiologies if new and atypical neurological symptoms occur in a HAM/TSP patient.

## 5. Therapy-Related Neurological Complications in ATL

### 5.1. Drug-Related Neurological Disorders

The effectiveness and availability of the treatment are limited in ATL. Besides clinical trials, the preferred regimens in first-line are dose-adjusted EPOCH (etoposide, prednisone, vincristine, cyclophosphamide, doxorubicin), brentuximab vedotin + CHP (cyclophosphamide, doxorubicin, prednisone), and zidovudine and interferon [57]. CHOP (cyclophosphamide, doxorubicin, vincristine, prednisone) is preferred for patients who are unable to tolerate intensive treatment [57]. In Japan, aggressive ATL is treated with VCAP/AMP/VECP (vincristine, cyclophosphamide, doxorubicin, prednisone/doxorubicin, ranimustine, prednisone/vindesine, etoposide, carboplatin, prednisone), which has higher effectiveness but also more side effects [91,92]. Since ranimustine and vindesine are unavailable outside of Japan, a modified VCAP/AMP/VECP regimen that uses vincristine instead of vindesine and excludes ranimustine can be adopted [92]. Monoclonal antibodies in monotherapy, such as mogamulizumab and brentuximab vedotin, lenalidomide, and other chemotherapy regimens are recommended as second-line or subsequent therapy [57]. As previously mentioned, CNS prophylaxis with regimens that include high-dose methotrexate or intrathecal methotrexate is indicated for all patients with acute or lymphoma-type ATL [57].

Most of the drugs used in ATL have the potential for both central and peripheral nervous system toxicity. We strongly advise consulting the summary of product characteristics when sudden neurological symptoms occur. Several drugs used in ATL, including vinca alkaloids (vincristine, vinorelbine, vindesine), platinum, cytarabine, etoposide, gemcitabine, ifosfamide, lenalidomide, and brentuximab vedotin, can cause peripheral neuropathy [39]. Methotrexate has certain particularities, causing acute, subacute, and chronic neurotoxicity [39]. Intrathecal methotrexate and intravenous high-dose methotrexate can result in stroke-like syndrome, with short-time neurological deficits, aphasia, encephalopathy, and seizures that resolve spontaneously in days [39]. Repeated administrations of high-dose methotrexate can lead to leukoencephalopathy after months or years, with cognitive impairment and personality disorders [39]. Intrathecal methotrexate can cause aseptic meningitis and transverse myelopathy [39]. After an extensive search in the literature, we did not find any cases of ATL with neurological toxicities. However, we considered it important to include them as possible factors of neurological manifestations.

### 5.2. Neurological Complications after Allogeneic Stem Cell Transplantation

Allo-HSCT remains a possible curative approach in aggressive-type ATL, especially when performed in complete response [8,92]. However, transplant and disease-related mortality are high [8,92]. Neurological complications are a common and life-threatening event after allo-HSCT. They are correlated with the conditioning regimen, immunosuppressive therapy such as calcineurin inhibitors, antibiotics, bone marrow aplasia with infectious and hemorrhagic risks, metabolic complications such as uremia and hepatocytolysis, and immune-mediated complications brought by graft-versus-host disease [40,41]. A study of 971 patients with hematological diseases who underwent allo-HSCT reported neurological complications in 132 patients [40]. Most of them experienced CNS neurological complications, such as stroke, posterior reversible encephalopathy syndrome, encephalopathy, isolated seizures, headache, and myelopathy [40]. The peripheral nervous system complications reported by the same study were peripheral neuropathy, myopathy, and neuromuscular junction disorders [40].

Data about neurological complications after allo-HSCT in ATL is scarce. Hirano et al., reported three cases of chronic inflammatory demyelinating polyneuropathy that developed after allo-HSCT for ATL. The symptoms included sensory loss, muscle weakness, and an absence of tendon reflex. They appeared at an interval of one month to seven years after transplantation, and all cases were associated with acute or chronic GVHD. Studies on nerve conduction revealed no electrical conduction on various nerves, and CSF analysis revealed albuminocytologic dissociation [93].

## 6. Hypercalcemic Crisis

Hypercalcemic crisis is a rare and potentially fatal complication of hypercalcemia that occurs when the albumin-corrected serum calcium level exceeds 14 mg/dL [94]. The severity of neurological complications varies, ranging from minor neuromuscular symptoms to cognitive impairment with confusion, poor concentration, personality changes, and even coma [94]. There are many causes of the hypercalcemic crisis, including hematological malignancies, such as multiple myeloma and lymphoma [94].

The pathogenesis of hypercalcemia in ATL patients was linked to increased activity of macrophage colony-stimulating factor, differentiation of hematopoietic precursor cells to osteoclasts via the receptor activator of nuclear factor kappa-B ligand on the ATL cells, ATL infiltration into the bone marrow, elevated levels of interleukin 1 and parathyroid hormone-related protein [95]. Approximately 70% of ATL patients acquire hypercalcemia over the course of the disease [96]. Hypercalcemic crises, on the other hand, are rarely observed. According to the literature, neurologic symptoms in ATL patients with hypercalcemia include decreased alertness, paralytic ileus presenting as constipation, and bilateral facial nerve weakness [42,43,44]. In all three cases, the patients were diagnosed with an aggressive type of ATL, and two of them died within a month of being diagnosed [42,43].

## 7. CNS Infections

Patients with hematological diseases frequently exhibit immunodeficiency, particularly those who undergo allo-HCST [97]. *Aspergillus*, *Toxoplasma*, mucoromycetes, and John Cunningham (JC) virus are prevalent in these patients, while bacterial CNS infections are uncommon [98]. Symptomatology in hematological patients is distinctive in that it can be disguised by other illnesses or reduced as a result of an inadequate inflammatory response [98]. According to the Guidelines of the Infectious Diseases Working Party of the German Society of Hematology and Medical Oncology, any suspicion of CNS infection should be followed by investigations for confirmation of the diagnosis, such as neuroimaging (preferable MRI, if not possible–CT scan, rarely FDG-PET), CSF examination, and, if necessary, biopsy [98]. Antimicrobial treatment should be started quickly following the completion of CSF and blood cultures according to the criteria given elsewhere [98].

Pathogens that can induce CNS infections in ATL include fungi [45,99], parasites [46], viruses [47,100,101,102,103], and bacteria [45,104]. The diagnosis of CNS infection is often obtained after necropsy. Clinical, biochemical, and imagistic findings lack specificity and are typically attributed to the course of the disease [46,105,106]. Furthermore, the blood–brain barrier is disrupted in individuals with CNS lymphoma, favoring the hematological dissemination of microorganisms [105].

We will further summarize some of the particularities of CNS infections in ATL. However, other etiological factors reported in different of hematological disorders and immunodeficiencies should be taken into consideration in the case of atypical neurological symptoms in ATL patients. CNS aspergillosis is a severe fungal infection with a variable clinical presentation depending on the involved structure. In immunocompromised patients, it often occurs by hematological dissemination but also by direct extension in disseminated aspergillosis [107]. CNS aspergillosis can occur in both aggressive and indolent types of ATL [45,99]. The outcome is dismal, even for the indolent types [99]. *Toxoplasma gondii* is a protozoan that can cause encephalitis and, less frequently, myelitis. In ATL patients, myelitis can be mistaken for HAM/TSP. Maciel et al., reported a case of an ATL patient who experienced progressive weakness in the lower limbs that quickly progressed to paraplegia, which was first attributed to HAM/TSP. The diagnosis of *Toxoplasma gondii* meningoencephalitis and myelitis was established at necropsy [46]. Bacterial CNS infections are less common in patients with hematological disorders compared to other etiologies. Usually, these patients have additional risk factors, such as intraventricular devices or previous surgical interventions. We found two cases of bacterial CNS infections in ATL patients, one with nocardial brain abscesses and the other with *Enterococcus* spp. meningitis [45,104]. Progressive multifocal leukoencephalopathy (PML) is a severe demyelinating disorder caused by infection with JC virus. PML was described in indolent and aggressive-types ATL, as well as in asymptomatic HTLV-1 carriers [47,100,102,103]. The presence of PML in carriers leads to the question of whether HTLV-1 is involved in PML development independent of disease and treatment-related immunosuppression. An early study found that HTLV-1 infection activates the transcriptional potential of JC virus promoters in neural cells through Tax expression [108]. The clinical manifestations are determined by the involved areas. In all four cases, the MRI revealed numerous hyperintensity lesions in the subcortical white matter. Except for one case, no gadolinium enhancement was seen. In that case, the authors attributed the anomaly to ATL cell invasion in the CNS [100].

## 8. Conclusions

The management of ATL neurological complications can be highly challenging. A wide spectrum of etiological factors, including disease progression in the CNS and neurolymphomatosis, treatment, especially allo-HSCT, and infections, can lead to potentially lethal complications. Moreover, the diagnosis is often made post-mortem, as symptoms might be masked or mimicked by another condition. Physicians should be aware of neurological abnormalities that can occur in ATL patients because early detection and treatment may improve the outcome. Therefore, we believe that ATL patients require a multidisciplinary approach with a team that comprises at least a hematologist, neurologist, infectious disease specialist, and radiologist.

## Figures and Tables

**Table 2:** World Health Organization diagnosis criteria for HAM/TSP [71,76,77].

**Clinical Criteria**
a. Age and sex incidence Mostly sporadic and adult, cut sometimes familial, occasionally seen in childhood; females predominant. b. Onset This is usually insidious but may be sudden c. Main neurological manifestations Chronic spastic paraparesis which usually progresses slowly, sometimes remains static after initial progression Weakness of the lower limbs, more marked proximally Bladder disturbance is usually an early feature, and constipation usually occurs later; impotence or decreased libido is common Sensory symptoms such as tingling, pins and needles, burning, etc., are more prominent than objective physical signs Low lumbar pain with radiation to the legs is common Vibration sense is frequently impaired, proprioception less often affected Hyperreflexia of the lower limbs, often with clonus and Babinski’s sign Hyperreflexia of the upper limbs, and positive Hoffmann’s and Tromner’s signs frequent; weakness may be absent Exaggerated jaw jerk in some patients d. Less frequent neurological findings Cerebellar signs; optic atrophy; deafness; nystagmus; other cranial nerve deficits; hand tremor; absent, or depressed ankle jerk Convulsions, cognitive impairment, dementia or impaired consciousness are rare e. Other neurological manifestations which may be associated with HAM/TSP Muscular atrophy; fasciculation (rare); polymyositis; peripheral neuropathy; polyradiculopathy; cranial neuropathy; meningitis; encephalopathy f. Systemic non-neurological manifestations which may be associated with HAM/TSP Pulmonary alveolitis; uveitis; Sjögren’s syndrome; arthropathy; vasculitis; ichthyoses; cryoglobulinemia; monoclonal gammopathy; adult T-cell leukemia/lymphoma
**Laboratory Diagnosis**
Presence of HTLV-1 antibodies or antigen in blood and CSF CSF may show mild lymphocyte pleocytosis Lobulated lymphocytes may be present in blood and/or CSF Mild to moderate increase of protein may be present in CSF Viral isolation, when possible, from blood and/or CSF

Abbreviations: HAM/TSP = HTLV-1 associated myelopathy/tropical spastic paraparesis.

**Table 3:** Osame motor disability score [81].

Grade	Motor Disability
0	No walking or running abnormalities
1	Normal gait but runs slowly
2	Abnormal gait (stumbling, stiffness)
3	Unable to run
4	Needs handrail to climb stairs
5	Needs a cane (unilateral support) to walk
6	Needs bilateral support to walk
7	Can walk 5–10 m with bilateral support
8	Can walk 1–5 m with bilateral support
9	Cannot walk, but able to crawl
10	Cannot crawl, but able to move using arms
11	Cannot move around, but able to turn over in bed
12	Cannot turn over in bed
13	Cannot even move toes

**Table 4:** Classification criteria for disease activity [80].

Disease Activity	Clinical Course	Biomarkers	
		CSF Neopterin (pmol/mL)	CSF CXCL10 (pg/mL)
High	Rapid progressor	≥44	≥4400
Moderate	Slow progressor	6–43	320–4399
Low	Very slow progressor	≤5	<320

Abbreviations: CXCL10 = C-X-C motif chemokine 10. Adapted from Sato, T. et al., Proposal of Classification Criteria for HTLV-1-Associated Myelopathy/Tropical Spastic Paraparesis Disease Activity. Front Microbiol 2018, 9, 1651. Licensed under a Creative Commons Attribution-NonCommercial 4.0 International License. Copyright 2018 Sato, Yagishita, Tamaki, Inoue, Hasegawa, Nagasaka, Suzuki, Araya, Coler-Reilly, Hasegawa, Tsuboi, Takata and Yamano.

**Table 5:** Published cases of HAM/TSP associated with ATL.

No.	Author	Year	Country	Clinical Course			Type of HAM/TSP	Age (HAM/TSP Onset)	Type of ATL	Age (ATL Diagnosis)	Sex
1	Bartholomew et al. [11]	1986	Trinidad and Tobago	HAM/TSP	→ (16 years)	ATL	N/A	33	lymphoma	49	M
2	Kawai et al. [12]	1989	Japan	HAM/TSP	→ (25 years)	ATL	slow progressor	17	chronic	42	F
3	Yoshida et al. [13]	1989	Japan	HAM/TSP	→ N/A	ATL	N/A	N/A	chronic	60	F
4	Yoshida et al. [13]	1989	Japan	HAM/TSP	→ N/A	ATL	N/A	N/A	smoldering	57	F
5	Yoshida et al. [13]	1989	Japan	HAM/TSP	→ N/A	ATL	N/A	N/A	chronic	42	F
6	Murata et al. [14]	1990	Japan	HAM/TSP	→ 22 years	ATL	slow progressor	20	lymphoma	42	F
7	Yasui et al. [15]	1991	Japan	ATL	→ N/A	HAM/TSP	N/A	N/A	smoldering	60	M
8	Honma S et al. [16]	1991	Japan	HAM/TSP	→ 2 years	ATL	slow progressor	55	smoldering	57	M
9	Iannone et al. [17]	1992	USA	ATL	→ 10 years	HAM/TSP	N/A	59	smoldering	49	M
10	Ikeda et al. [18]	1993	Japan	ATL	+	HAM/TSP	N/A	36	smoldering	36	M
11	Furukawa Y et al. [19]	1995	Japan	HAM/TSP	→ 11 years	ATL	slow progressor	26	acute	47	F
12	Tamiya S et al. [20]	1995	Japan	HAM/TSP	→ N/A	ATL	N/A	N/A	lymphoma	53	F
13	Tamiya S et al. [20]	1995	Japan	HAM/TSP	→ N/A	ATL	N/A	N/A	lymphoma	56	F
14	Freitas V et al. [21]	1997	Brazil	HAM/TSP	→ 4 years	ATL	N/A	55	chronic	59	M
15	Kanno M et al. [22]	1998	Japan	ATL	→ 2 years	HAM/TSP	N/A	74	N/A	72	M
16	Goncalves DU et al. [23]	1999	Brazil	HAM/TSP	→ 3 years	ATL	N/A	24	smoldering	27	F
17	Kasahata N et al. [24]	2000	Japan	HAM/TSP	→ N/A	ATL	N/A	N/A	acute	35	M
18	Michael EJ et al. [25]	2002	Jamaica	HAM/TSP	→ N/A	ATL	N/A	N/A	acute	60	M
19	Farre L et al. [26]	2008	Brazil	ATL	+	HAM/TSP	N/A	16	smoldering	16	F
20	Umehara F et al. [27]	2008	Japan	ATL	+	HAM/TSP	N/A	55	chronic	55	F
21	Goncalves DU et al. [28]	2009	Brazil	HAM/TSP	→ 2 years	ATL	N/A	19	acute	21	M
22	Sharma et al. [29]	2018	Haiti	ATL	→ 4 years	HAM/TSP	N/A	28	N/A	24	F
23	Nakaya Y et al. [30]	2019	Japan	HAM/TSP	→ 24 years	ATL	rapid progressor	32	lymphoma	56	M
24	Takeda et al. [31]	2020	Japan	HAM/TSP	→ 20 years	ATL	slow progressor	35	acute	55	F
25	Sakamoto H et al. [32]	2021	Japan	HAM/TSP	→ 10 years	ATL	slow progressor	48	lymphoma	58	M
26	Tamaki K et al. [33]	2021	Japan	HAM/TSP	→ 5 months	ATL	rapid progressor	54	lymphoma	54	M

## Data Availability

Not applicable.
